# Crystal structure of poly[[(acetato-κ*O*){μ_3_-*N*-[(pyridin-4-yl)meth­yl]pyrazine-2-carboxamidato-κ^4^ *N*:*N* ^1^,*N* ^2^:*N* ^4^]copper(II)] dihydrate]: a metal–organic framework (MOF)^1^

**DOI:** 10.1107/S1600536814011520

**Published:** 2014-06-23

**Authors:** Dilovan S. Cati, Helen Stoeckli-Evans

**Affiliations:** a Debiopharm International S.A., Chemin Messidor 5-7, CP 5911, 1002 Lausanne, Switzerland; b Institute of Physics, University of Neuchâtel, rue Emile-Argand 11, CH-2000 Neuchâtel, Switzerland

**Keywords:** crystal structure, metal-organic framework, 10 (3) network topology, copper(II), pyrazine-2-carboxamide

## Abstract

The title compound, [Cu(C_11_H_9_N_4_O)(CH_3_CO_2_)]·2H_2_O (Cu*L*), is a hydrated copper acetate complex of the ligand *N*-[(pyridin-4-yl)meth­yl]pyrazine-2-carboxamide (H*L*). Complex Cu*L* has a metal–organic framework (MOF) structure with a 10 (3) network topology. The ligand coordinates in a bidentate and a bis-monodentate manner, bridging three equivalent Cu^II^ atoms *via* the pyridine N atom and the second pyrazine N atom. The Cu^II^ atom has a fivefold coordination sphere, CuN_4_O, being coordinated to three N atoms of the ligand and the acetate O atom in the equatorial plane and to the second pyrazine atom in the apical position. This gives rise to a fairly regular square-pyramidal geometry. In the crystal, the water mol­ecules are linked to each other and to the three-dimensional framework *via* O—H⋯O hydrogen bonds. There are also a number of C—H⋯O hydrogen bonds present within the framework.

## Abstract 2

The title compound, a hydrated copper acetate complex of the ligand *N*-[(pyridin-4-yl)methyl]pyrazine-2-carboxamide, has a metal-organic framework (MOF) structure with a 10 (3) network topology. The water molecules are located in the cavities of the framework and linked to it by O—H⋯O hydrogen bonds.

## Chemical context   

The ligand *N*-[(pyridin-4-yl)meth­yl]pyrazine-2-carboxamide (H*L*) is one of a series of ligands which were synthesized in order to study their coordination behaviour towards first-row transition metals (Cati, 2002 ▶; Cati *et al.*, 2004 ▶; Cati & Stoeckli-Evans, 2014 ▶). H*L* is expected to coordinate in a bidentate and possibly a monodentate manner, with eventual bridging of metal atoms to construct two- or three-dimensional networks. A excellent review on the subject of coordination polymers and network structures has been published by Batten *et al.* (2009 ▶).
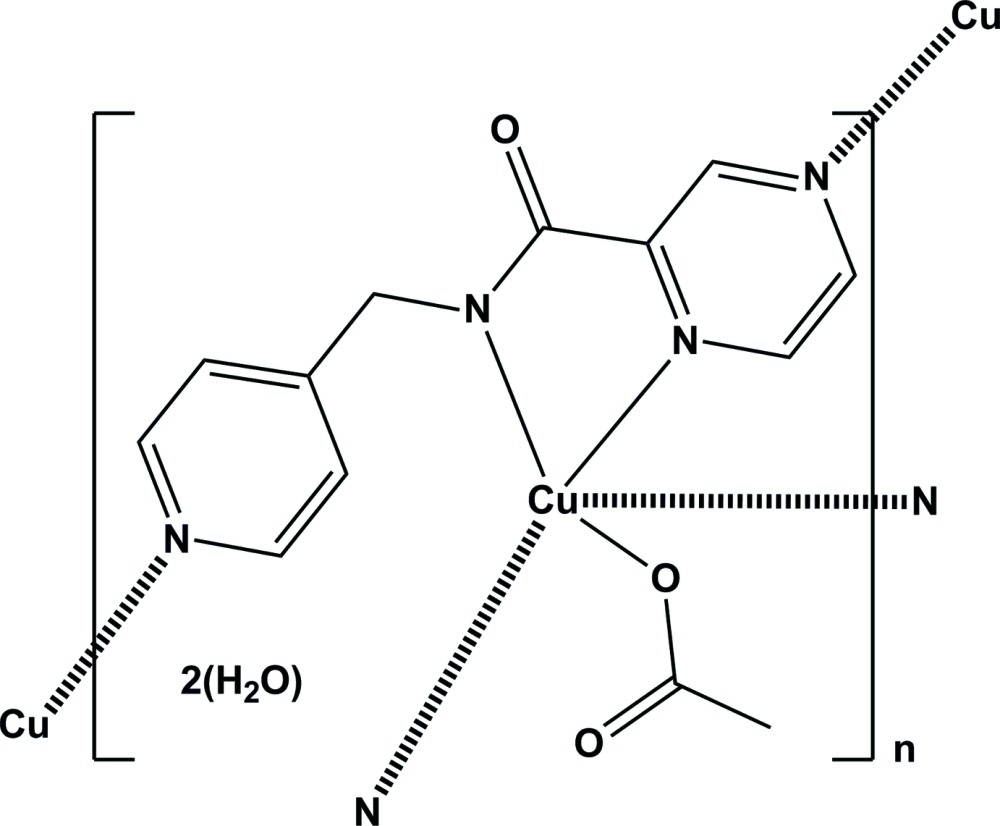


## Structural commentary   

**Figure 1:**
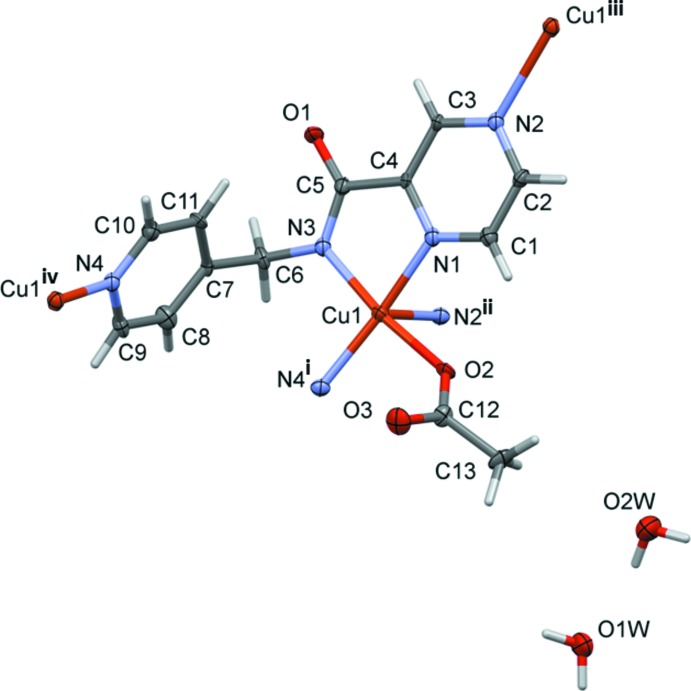
A view of the asymmetric unit of complex Cu*L*, with atom labelling [symmetry codes: (i) *x*, −*y*, *z* − 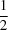; (ii) *x* − 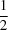, −*y* + 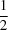, *z* − 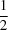; (iii) *x* + 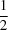, −*y* + 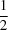, *z* + 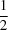; (iv) *x*, −*y*, *z* + 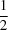]. Displacement ellipsoids are drawn at the 50% probability level.

**Figure 2:**
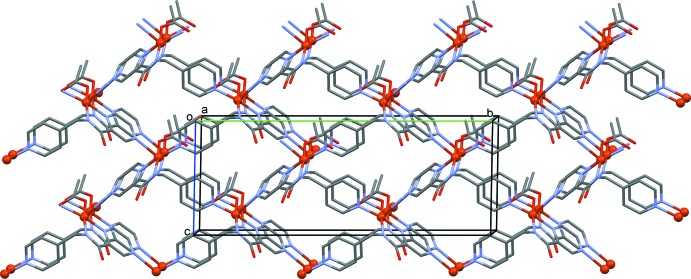
A view along the *a* axis of the metal–organic framework (MOF) structure of complex Cu*L*. Solvent water mol­ecules and H atoms have been omitted for clarity.

The title compound, Cu*L*, is a copper acetate complex of the ligand *N*-[(pyridin-4-yl)meth­yl]pyrazine-2-carboxamide (H*L*) [Cati & Stoeckli-Evans, 2014 ▶]. In complex Cu*L* the ligand coordinates in a bidentate and a bis-monodentate manner, so bridging three equivalent copper atoms (Fig. 1 ▶). This gives rise to the formation of a three-dimensional coordination polymer, or MOF (metal–organic framework) structure, as shown in Fig. 2 ▶. The copper⋯copper distances are 7.156 (2) Å *via* the bridging pyrazine ring (Cu1⋯Cu1^iii^) and 7.420 (2) Å *via* the pyridine N atom Cu1⋯Cu1^iv^; see Fig. 1 ▶). Atom Cu1 has a fivefold coordination sphere, CuN_4_O, with three N atoms (N1, N3 and N4^i^) and the acetate O atom, O2, in the equatorial plane and the second pyrazine N atom, N2^ii^, in the apical position [Fig. 2 ▶; symmetry codes: (i) *x*, −*y*, *z* − 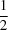; (ii) *x* − 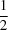, −*y* + 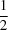, *z* − 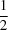]. The apical Cu1—N2 bond distance of 2.393 (3) Å is considerably longer that the Cu1–N1, Cu1—N3 and Cu1—N4 bond lengths [2.003 (8), 1.964 (9) and 1.993 (7) Å, respectively], and the Cu1—O2 bond length [1.947 (7) Å] in the equatorial plane. Bond angles O2—Cu1—N3 and N4—Cu1—N1 are 172.2 (3) and 170.6 (3)°, respectively, and this leads to a perfect square-pyramidal geometry with τ = 0.03 (τ = 0 square-pyramidal; τ = 1 trigonal-bipyramidal; Addison *et al.*, 1984 ▶). The pyridine ring is inclined to the pyrazine ring by 79.6 (5)° compared to 84.33 (12)° in the free ligand (Cati & Stoeckli-Evans, 2014 ▶). The bond distances and angles are normal when compared with geometrical parameters of related copper(II) complexes in the Cambridge Structural Database (Version 5.35, last update November 2013; Allen, 2002 ▶), and are similar to those observed in the mononuclear copper(II) acetate complex of the analogous ligand *N*-[(pyridin-2-yl)methyl]pyrazine-2-carboxamide (Moh­a­­madou *et al.*, 2012 ▶). The title compound crystallizes with two solvent water mol­ecules per asymmetric unit.

## Supra­molecular features   

**Figure 3:**
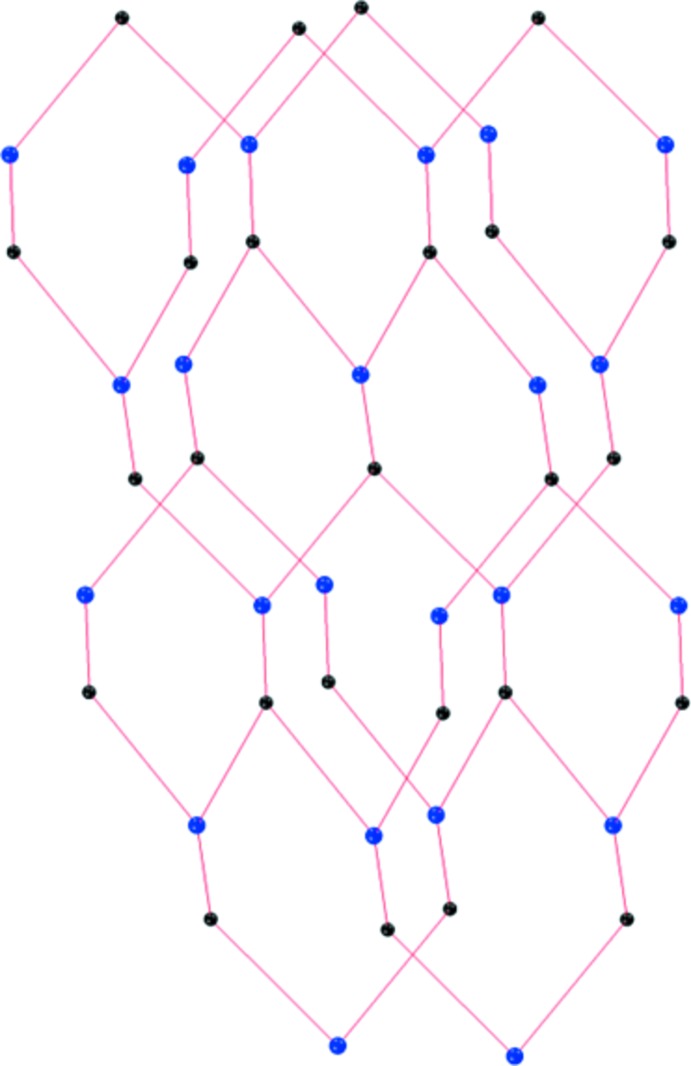
A view of the 10 (3) network topology of the title metal–organic framework (MOF) structure, illustrating the 3-connected three-dimensional nets with ten-membered rings.

The three-dimensional network of the title MOF structure has a 10 (3) network topology (Fig. 3 ▶). It is one of the most commonly encountered 3-connected three-dimensional nets with ten-membered rings (Wells, 1984 ▶). It is a cubic (10,3)-a net, also known as the srs (SrSi_2_) net, which is chiral [note that the Flack *x* parameter = −0.01 (3)]. Such structures contain fourfold helices along the three axes all of the same hand (Batten *et al.*, 2009 ▶).

**Figure 4:**
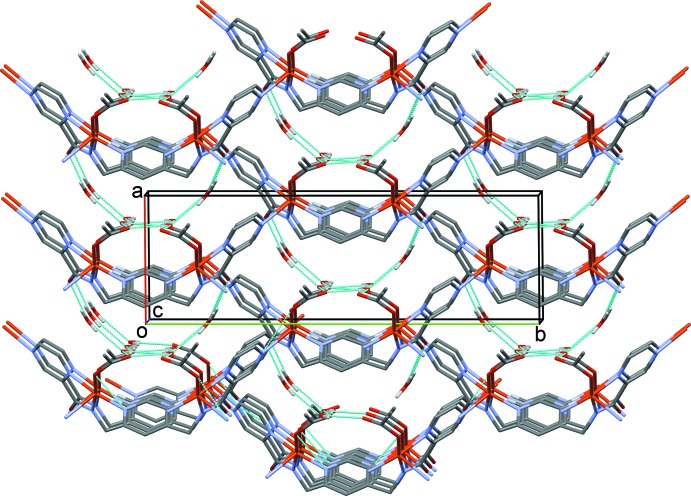
A view along the *c* axis of the crystal packing of complex Cu*L*, with the hydrogen bonds involving the water mol­ecules shown as dashed lines (see Table 1 ▶ for details; H atoms not involved in these hydrogen bonding have been omitted for clarity).

In the crystal of Cu*L*, the water mol­ecules are located in the cavities of the MOF structure. They are hydrogen bonded to one another and to the ligand and acetate carbonyl O atoms (Table 1 ▶ and Fig. 4 ▶). There are also a number of C—H⋯O hydrogen bonds present within the framework (Table 1 ▶).

## Database survey   

A search of the Cambridge Structural Database (Version 5.35, last update November 2013; Allen, 2002 ▶) indicated that no complexes of the ligand H*L* have been described previously. The analogous ligand *N*-[(pyridin-2-yl)methyl]pyrazine-2-carboxamide has been described as well as a number of metal complexes. These include the mononuclear copper acetate complex (Mohamadou *et al.*, 2012 ▶). Here this ligand coordin­ates in a tridentate manner but in a number of other complexes it coordinates in a bis-monodentate manner *via* the pyridine N atom and a pyrazine N atom; for example, in two polymeric mercury chloride complexes (Khavasi *et al.*, 2010 ▶), and a polymeric silver tetra­fluoro­borate complex (Hellyer *et al.*, 2009 ▶).

## Synthesis and crystallization   

The synthesis of the ligand *N*-[(pyridin-4-yl)meth­yl]pyrazine-2-carboxamide (H*L*) has been described elsewhere (Cati, 2002 ▶; Cati & Stoeckli-Evans, 2014 ▶). Complex Cu*L* was prepared by adding Cu(acetate)_2_·H_2_O (64 mg, 0.318 mmol) to a hot solution (323 K) of H*L* (68 mg, 0.318 mmol) in dry methanol (25 ml). In 2 min a precipitate appeared and heating was stopped and the mixture stirred as the temperature decreased to room temperature. After 30 min the precipitate was filtered off and washed with dry methanol. It was then dissolved in a mixture of water (12 ml) and methanol (15 ml) and stirred with warming to between 313 to 323 K for 15 min. The resulting blue solution was allowed to stand at room temperature and yielded blue crystals in a few days [yield 72 mg, 61%]. Analysis for C_13_H_12_CuN_4_O_3_·2(H_2_O) (*M_r_* = 371.84). Calculated (%): C 41.99, H 4.34, N 15.07. Found: C 42.17, H 4.33, N 14.75.

## Refinement   

Crystal data, data collection and structure refinement details are summarized in Table 2 ▶. The water H atoms were located in difference Fourier maps were refined with distance restraints: O—H = 0.84 (2) and H⋯H = 1.35 (2) Å with *U*_iso_(H) = 1.5U_eq_(O). The C-bound H atoms were included in calculated positions and treated as riding atoms: C—H = 0.95 Å with *U*_iso_(H) = 1.2*U*_eq_(C).

## Supplementary Material

Crystal structure: contains datablock(s) CuL. DOI: 10.1107/S1600536814011520/hb0008sup1.cif

Structure factors: contains datablock(s) CuL2. DOI: 10.1107/S1600536814011520/hb0008CuL2sup2.hkl

CCDC reference: 1004264

Additional supporting information:  crystallographic information; 3D view; checkCIF report

## Figures and Tables

**Table 1:** Hydrogen-bond geometry (Å, °)

*D*—H⋯*A*	*D*—H	H⋯*A*	*D*⋯*A*	*D*—H⋯*A*
O1*W*—H1*WA*⋯O3^i^	0.84 (3)	2.13 (5)	2.908 (9)	154 (9)
O1*W*—H1*WB*⋯O3^iii^	0.84 (3)	2.23 (5)	2.964 (10)	146 (8)
O2*W*—H2*WA*⋯O1*W*	0.86 (3)	2.11 (4)	2.951 (10)	165 (10)
O2*W*—H2*WB*⋯O1^iv^	0.85 (3)	2.20 (3)	3.033 (8)	169 (10)
C2—H2⋯O2^v^	0.95	2.37	2.987 (13)	123
C8—H8⋯O3^vi^	0.95	2.57	3.364 (11)	141
C9—H9⋯O2*W* ^vii^	0.95	2.50	3.358 (12)	151

Symmetry codes: (i) 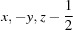; (iii) 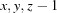; (iv) 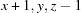; (v) 
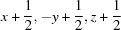
; (vi) 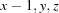; (vii) 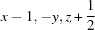.

**Table 2:** Experimental details

Crystal data	
Chemical formula	[Cu(C_11_H_9_N_4_O)(C_2_H_3_O_2_)]·2H_2_O
*M*_r_	371.84
Crystal system, space group	Monoclinic, *C* *c*
Temperature (K)	153
*a*, *b*, *c* (Å)	7.8256 (12), 22.331 (2), 8.9976 (13)
β (°)	110.040 (16)
*V* (Å^3^)	1477.2 (4)
*Z*	4
Radiation type	Mo *K*α
μ (mm^−1^)	1.51
Crystal size (mm)	0.40 × 0.30 × 0.30

Data collection	
Diffractometer	Stoe *IPDS* I
Absorption correction	Multi-scan (*MULscanABS* in *PLATON*; Spek, 2009 ▶)
*T*_min_, *T*_max_	0.979, 1.000
No. of measured, independent and observed [*I* > 2σ(*I*)] reflections	5730, 2705, 1778
*R*_int_	0.070
(sin θ/λ)_max_ (Å^−1^)	0.615

Refinement	
*R*[*F* ^2^ > 2σ(*F* ^2^)], *wR*(*F* ^2^), *S*	0.040, 0.072, 0.78
No. of reflections	2705
No. of parameters	203
No. of restraints	8
H-atom treatment	H-atom parameters constrained
Δρ_max_, Δρ_min_ (e Å^−3^)	0.40, −0.59
Absolute structure	Flack *x* determined using 665 quotients [(*I* ^+^)−(*I* ^−^)]/[(*I* ^+^)+(*I* ^−^)] (Parsons & Flack, 2004 ▶)
Absolute structure parameter	−0.01 (3)

Computer programs: *EXPOSE* in *IPDSI*, *CELL* and *INTEGRATE* in *IPDSI* (Stoe & Cie, 2004 ▶), *SHELXS97* and *SHELXL2013* (Sheldrick, 2008 ▶), *Mercury* (Macrae *et al.*, 2008 ▶), *PLATON* (Spek, 2009 ▶) and *publCIF* (Westrip, 2010 ▶).

## supplementary crystallographic information

### Crystal data

**Table:**

[Cu(C_11_H_9_N_4_O)(C_2_H_3_O_2_)]·2H_2_O	*F*(000) = 764
*M**_r_* = 371.84	*D*_x_ = 1.672 Mg m^−^^3^
Monoclinic, *C**c*	Mo *K*α radiation, λ = 0.71073 Å
Hall symbol: -P 2yn	Cell parameters from 2705 reflections
*a* = 7.8256 (12) Å	θ = 2.1–26.1°
*b* = 22.331 (2) Å	µ = 1.51 mm^−^^1^
*c* = 8.9976 (13) Å	*T* = 153 K
β = 110.040 (16)°	Block, turquoise blue
*V* = 1477.2 (4) Å^3^	0.40 × 0.30 × 0.30 mm
*Z* = 4	

### Data collection

**Table:**

Stoe IPDS I diffractometer	2705 independent reflections
Radiation source: fine-focus sealed tube	1778 reflections with *I* > 2σ(*I*)
Plane graphite monochromator	*R*_int_ = 0.070
φ rotation scans	θ_max_ = 25.9°, θ_min_ = 2.9°
Absorption correction: multi-scan (*MULscanABS* in *PLATON*; Spek, 2009)	*h* = −9→9
*T*_min_ = 0.979, *T*_max_ = 1.000	*k* = −27→26
5730 measured reflections	*l* = −11→10

### Refinement

**Table:**

Refinement on *F*^2^	Secondary atom site location: difference Fourier map
Least-squares matrix: full	Hydrogen site location: mixed
*R*[*F*^2^ > 2σ(*F*^2^)] = 0.040	H-atom parameters constrained
*wR*(*F*^2^) = 0.072	*w* = 1/[σ^2^(*F*_o_^2^) + (0.007*P*)^2^] where *P* = (*F*_o_^2^ + 2*F*_c_^2^)/3
*S* = 0.78	(Δ/σ)_max_ < 0.001
2705 reflections	Δρ_max_ = 0.40 e Å^−^^3^
203 parameters	Δρ_min_ = −0.59 e Å^−^^3^
8 restraints	Absolute structure: Flack *x* determined using 665 quotients [(*I*^+^)-(*I*^-^)]/[(*I*^+^)+(*I*^-^)] (Parsons & Flack, 2004)
Primary atom site location: structure-invariant direct methods	Absolute structure parameter: −0.01 (3)

### Special details

**Table:**

Geometry. All e.s.d.'s (except the e.s.d. in the dihedral angle between two l.s. planes) are estimated using the full covariance matrix. The cell e.s.d.'s are taken into account individually in the estimation of e.s.d.'s in distances, angles and torsion angles; correlations between e.s.d.'s in cell parameters are only used when they are defined by crystal symmetry. An approximate (isotropic) treatment of cell e.s.d.'s is used for estimating e.s.d.'s involving l.s. planes.

### Fractional atomic coordinates and isotropic or equivalent isotropic displacement parameters (Å^2^)

**Table:**

	*x*	*y*	*z*	*U*_iso_*/*U*_eq_	
Cu1	0.44720 (19)	0.13211 (4)	0.83884 (18)	0.0155 (2)	
O1	0.3072 (8)	0.1995 (2)	1.2004 (7)	0.0248 (16)	
O2	0.5973 (10)	0.1326 (3)	0.7054 (9)	0.0169 (15)	
O3	0.7386 (8)	0.0439 (3)	0.7464 (8)	0.0266 (15)	
N1	0.5958 (11)	0.1988 (3)	0.9695 (9)	0.0155 (9)	
N2	0.7658 (12)	0.2933 (3)	1.1646 (9)	0.0155 (9)	
N3	0.3109 (12)	0.1421 (4)	0.9844 (11)	0.0155 (9)	
N4	0.3255 (10)	−0.0570 (3)	1.2361 (9)	0.0155 (9)	
C1	0.7378 (15)	0.2255 (5)	0.9510 (12)	0.018 (3)	
H1	0.7839	0.2120	0.8720	0.022*	
C2	0.8206 (16)	0.2742 (5)	1.0483 (12)	0.018 (3)	
H2	0.9191	0.2940	1.0304	0.021*	
C3	0.6208 (15)	0.2647 (4)	1.1831 (13)	0.015 (2)	
H3	0.5771	0.2774	1.2644	0.018*	
C4	0.5351 (15)	0.2174 (5)	1.0852 (11)	0.014 (2)	
C5	0.3707 (11)	0.1848 (3)	1.0932 (10)	0.0146 (19)	
C6	0.1512 (13)	0.1078 (4)	0.9801 (12)	0.015 (2)	
H6A	0.0774	0.0987	0.8689	0.018*	
H6B	0.0757	0.1320	1.0262	0.018*	
C7	0.2058 (11)	0.0497 (3)	1.0721 (11)	0.0124 (17)	
C8	0.1360 (11)	−0.0045 (4)	1.0059 (11)	0.020 (2)	
H8	0.0425	−0.0061	0.9053	0.024*	
C9	0.2044 (13)	−0.0571 (4)	1.0884 (13)	0.020 (2)	
H9	0.1638	−0.0944	1.0381	0.024*	
C10	0.3872 (11)	−0.0036 (3)	1.3001 (11)	0.019 (2)	
H10	0.4747	−0.0028	1.4039	0.023*	
C11	0.3310 (12)	0.0503 (4)	1.2239 (10)	0.016 (2)	
H11	0.3775	0.0871	1.2748	0.019*	
C12	0.6927 (13)	0.0907 (5)	0.6705 (13)	0.016 (2)	
C13	0.7531 (12)	0.1058 (4)	0.5323 (10)	0.024 (2)	
H13A	0.7724	0.0687	0.4820	0.035*	
H13B	0.8669	0.1285	0.5701	0.035*	
H13C	0.6591	0.1298	0.4552	0.035*	
O1W	0.7662 (12)	0.0650 (3)	0.0791 (10)	0.0313 (19)	
H1WA	0.764 (17)	0.042 (4)	0.151 (8)	0.047*	
H1WB	0.752 (15)	0.044 (4)	−0.002 (7)	0.047*	
O2W	0.9960 (9)	0.1546 (3)	0.2989 (8)	0.0358 (18)	
H2WA	0.925 (10)	0.134 (4)	0.222 (8)	0.054*	
H2WB	1.072 (10)	0.169 (4)	0.261 (10)	0.054*	

### Atomic displacement parameters (Å^2^)

**Table:**

	*U*^11^	*U*^22^	*U*^33^	*U*^12^	*U*^13^	*U*^23^
Cu1	0.0184 (5)	0.0119 (4)	0.0183 (5)	−0.0019 (8)	0.0092 (4)	−0.0025 (8)
O1	0.032 (4)	0.021 (3)	0.032 (4)	−0.011 (3)	0.025 (4)	−0.013 (3)
O2	0.023 (4)	0.008 (3)	0.023 (4)	0.000 (3)	0.012 (3)	−0.004 (3)
O3	0.025 (4)	0.019 (3)	0.037 (5)	0.009 (3)	0.013 (3)	0.005 (3)
N1	0.018 (2)	0.0133 (18)	0.018 (2)	−0.0018 (16)	0.009 (2)	0.0013 (17)
N2	0.018 (2)	0.0133 (18)	0.018 (2)	−0.0018 (16)	0.009 (2)	0.0013 (17)
N3	0.018 (2)	0.0133 (18)	0.018 (2)	−0.0018 (16)	0.009 (2)	0.0013 (17)
N4	0.018 (2)	0.0133 (18)	0.018 (2)	−0.0018 (16)	0.009 (2)	0.0013 (17)
C1	0.017 (6)	0.017 (5)	0.020 (6)	−0.004 (4)	0.007 (5)	−0.002 (5)
C2	0.019 (6)	0.022 (6)	0.016 (6)	−0.003 (5)	0.011 (5)	−0.001 (5)
C3	0.018 (6)	0.014 (5)	0.016 (6)	−0.005 (4)	0.012 (5)	−0.001 (4)
C4	0.023 (6)	0.010 (5)	0.012 (5)	−0.005 (4)	0.010 (5)	0.001 (4)
C5	0.015 (5)	0.016 (4)	0.014 (5)	−0.002 (3)	0.006 (4)	0.001 (4)
C6	0.013 (6)	0.010 (5)	0.021 (6)	−0.002 (4)	0.004 (5)	0.003 (4)
C7	0.011 (4)	0.009 (4)	0.018 (5)	0.002 (3)	0.006 (4)	0.006 (4)
C8	0.012 (5)	0.020 (4)	0.027 (6)	0.001 (4)	0.005 (4)	0.000 (4)
C9	0.020 (5)	0.012 (5)	0.028 (7)	−0.004 (4)	0.009 (5)	0.002 (4)
C10	0.024 (6)	0.017 (4)	0.017 (6)	0.000 (3)	0.009 (5)	0.002 (4)
C11	0.023 (5)	0.007 (4)	0.018 (5)	−0.003 (4)	0.008 (5)	−0.004 (4)
C12	0.007 (5)	0.022 (6)	0.019 (6)	0.001 (4)	0.004 (5)	0.001 (5)
C13	0.019 (5)	0.035 (5)	0.022 (6)	0.004 (4)	0.014 (5)	−0.003 (4)
O1W	0.047 (5)	0.019 (4)	0.033 (5)	−0.002 (4)	0.019 (5)	0.001 (3)
O2W	0.042 (5)	0.038 (4)	0.034 (5)	−0.007 (3)	0.022 (4)	−0.006 (3)

### Geometric parameters (Å, º)

**Table:**

Cu1—O2	1.947 (7)	C3—H3	0.9500
Cu1—N3	1.964 (9)	C4—C5	1.500 (13)
Cu1—N4^i^	1.993 (7)	C6—C7	1.520 (11)
Cu1—N1	2.003 (8)	C6—H6A	0.9900
Cu1—N2^ii^	2.393 (8)	C6—H6B	0.9900
O1—C5	1.270 (9)	C7—C8	1.376 (11)
O2—C12	1.300 (12)	C7—C11	1.382 (12)
O3—C12	1.232 (11)	C8—C9	1.395 (13)
N1—C1	1.323 (13)	C8—H8	0.9500
N1—C4	1.349 (12)	C9—H9	0.9500
N2—C2	1.330 (13)	C10—C11	1.381 (11)
N2—C3	1.360 (13)	C10—H10	0.9500
N2—Cu1^iii^	2.393 (8)	C11—H11	0.9500
N3—C5	1.332 (11)	C12—C13	1.512 (12)
N3—C6	1.455 (13)	C13—H13A	0.9800
N4—C10	1.341 (10)	C13—H13B	0.9800
N4—C9	1.342 (13)	C13—H13C	0.9800
N4—Cu1^iv^	1.992 (7)	O1W—H1WA	0.84 (3)
C1—C2	1.405 (14)	O1W—H1WB	0.84 (3)
C1—H1	0.9500	O2W—H2WA	0.86 (3)
C2—H2	0.9500	O2W—H2WB	0.85 (3)
C3—C4	1.393 (13)		
			
O2—Cu1—N3	172.2 (3)	O1—C5—N3	127.8 (8)
O2—Cu1—N4^i^	90.7 (3)	O1—C5—C4	118.5 (8)
N3—Cu1—N4^i^	97.0 (3)	N3—C5—C4	113.7 (8)
O2—Cu1—N1	90.4 (3)	N3—C6—C7	110.9 (8)
N3—Cu1—N1	82.1 (4)	N3—C6—H6A	109.5
N4^i^—Cu1—N1	170.6 (3)	C7—C6—H6A	109.5
O2—Cu1—N2^ii^	86.5 (3)	N3—C6—H6B	109.5
N3—Cu1—N2^ii^	91.3 (3)	C7—C6—H6B	109.5
N4^i^—Cu1—N2^ii^	101.5 (3)	H6A—C6—H6B	108.0
N1—Cu1—N2^ii^	87.8 (2)	C8—C7—C11	118.6 (8)
C12—O2—Cu1	131.5 (6)	C8—C7—C6	121.3 (8)
C1—N1—C4	119.4 (8)	C11—C7—C6	120.1 (8)
C1—N1—Cu1	127.3 (7)	C7—C8—C9	119.2 (9)
C4—N1—Cu1	113.3 (7)	C7—C8—H8	120.4
C2—N2—C3	116.9 (9)	C9—C8—H8	120.4
C2—N2—Cu1^iii^	117.4 (7)	N4—C9—C8	122.6 (9)
C3—N2—Cu1^iii^	125.3 (7)	N4—C9—H9	118.7
C5—N3—C6	118.6 (9)	C8—C9—H9	118.7
C5—N3—Cu1	115.9 (6)	N4—C10—C11	123.8 (8)
C6—N3—Cu1	125.5 (7)	N4—C10—H10	118.1
C10—N4—C9	117.0 (7)	C11—C10—H10	118.1
C10—N4—Cu1^iv^	120.3 (6)	C10—C11—C7	118.6 (8)
C9—N4—Cu1^iv^	121.4 (6)	C10—C11—H11	120.7
N1—C1—C2	119.9 (10)	C7—C11—H11	120.7
N1—C1—H1	120.0	O3—C12—O2	124.0 (9)
C2—C1—H1	120.0	O3—C12—C13	122.0 (9)
N2—C2—C1	122.3 (10)	O2—C12—C13	113.9 (8)
N2—C2—H2	118.9	C12—C13—H13A	109.5
C1—C2—H2	118.9	C12—C13—H13B	109.5
N2—C3—C4	121.3 (10)	H13A—C13—H13B	109.5
N2—C3—H3	119.3	C12—C13—H13C	109.5
C4—C3—H3	119.3	H13A—C13—H13C	109.5
N1—C4—C3	120.1 (10)	H13B—C13—H13C	109.5
N1—C4—C5	115.0 (8)	H1WA—O1W—H1WB	107 (4)
C3—C4—C5	124.9 (9)	H2WA—O2W—H2WB	104 (4)
			
C4—N1—C1—C2	−2.0 (14)	N1—C4—C5—N3	0.4 (12)
Cu1—N1—C1—C2	176.4 (8)	C3—C4—C5—N3	178.4 (10)
C3—N2—C2—C1	−1.9 (15)	C5—N3—C6—C7	−95.5 (10)
Cu1^iii^—N2—C2—C1	172.1 (9)	Cu1—N3—C6—C7	85.8 (10)
N1—C1—C2—N2	2.8 (17)	N3—C6—C7—C8	−128.6 (9)
C2—N2—C3—C4	0.5 (14)	N3—C6—C7—C11	49.7 (12)
Cu1^iii^—N2—C3—C4	−173.0 (8)	C11—C7—C8—C9	−5.2 (12)
C1—N1—C4—C3	0.5 (14)	C6—C7—C8—C9	173.2 (8)
Cu1—N1—C4—C3	−178.0 (8)	C10—N4—C9—C8	−3.5 (12)
C1—N1—C4—C5	178.7 (9)	Cu1^iv^—N4—C9—C8	−171.0 (7)
Cu1—N1—C4—C5	0.1 (11)	C7—C8—C9—N4	5.7 (13)
N2—C3—C4—N1	0.2 (16)	C9—N4—C10—C11	1.1 (11)
N2—C3—C4—C5	−177.7 (9)	Cu1^iv^—N4—C10—C11	168.8 (6)
C6—N3—C5—O1	1.8 (14)	N4—C10—C11—C7	−0.9 (12)
Cu1—N3—C5—O1	−179.4 (7)	C8—C7—C11—C10	2.9 (12)
C6—N3—C5—C4	−179.5 (9)	C6—C7—C11—C10	−175.5 (8)
Cu1—N3—C5—C4	−0.7 (10)	Cu1—O2—C12—O3	−17.2 (15)
N1—C4—C5—O1	179.2 (8)	Cu1—O2—C12—C13	166.2 (7)
C3—C4—C5—O1	−2.8 (15)		

Symmetry codes: (i) *x*, −*y*, *z*−1/2; (ii) *x*−1/2, −*y*+1/2, *z*−1/2; (iii) *x*+1/2, −*y*+1/2, *z*+1/2; (iv) *x*, −*y*, *z*+1/2.

### Hydrogen-bond geometry (Å, º)

**Table:**

*D*—H···*A*	*D*—H	H···*A*	*D*···*A*	*D*—H···*A*
O1*W*—H1*WA*···O3^i^	0.84 (3)	2.13 (5)	2.908 (9)	154 (9)
O1*W*—H1*WB*···O3^v^	0.84 (3)	2.23 (5)	2.964 (10)	146 (8)
O2*W*—H2*WA*···O1*W*	0.86 (3)	2.11 (4)	2.951 (10)	165 (10)
O2*W*—H2*WB*···O1^vi^	0.85 (3)	2.20 (3)	3.033 (8)	169 (10)
C2—H2···O2^iii^	0.95	2.37	2.987 (13)	123
C8—H8···O3^vii^	0.95	2.57	3.364 (11)	141
C9—H9···O2*W*^viii^	0.95	2.50	3.358 (12)	151

Symmetry codes: (i) *x*, −*y*, *z*−1/2; (iii) *x*+1/2, −*y*+1/2, *z*+1/2; (v) *x*, *y*, *z*−1; (vi) *x*+1, *y*, *z*−1; (vii) *x*−1, *y*, *z*; (viii) *x*−1, −*y*, *z*+1/2.
